# Cooking outdoors or with cleaner fuels does not increase malarial risk in children under 5 years: a cross-sectional study of 17 sub-Saharan African countries

**DOI:** 10.1186/s12936-022-04152-3

**Published:** 2022-04-27

**Authors:** Katherine E. Woolley, Suzanne E. Bartington, Francis D. Pope, Sheila M. Greenfield, Lucy S. Tusting, Malcolm J. Price, G. Neil Thomas

**Affiliations:** 1 Institute of Applied Health Research, University of Birmingham, Edgbaston, Birmingham, UK; 2 School of Geography, Earth and Environmental Sciences, University of Birmingham, Edgbaston, Birmingham, UK; 3 Department of Disease Control, London School of Hygiene and Tropical Medicine, London, UK; 4 Centre on Climate Change and Planetary Health, London School of Hygiene & Tropical Medicine, London, UK; 5 NIHR Birmingham Biomedical Research Centre, University Hospitals Birmingham NHS Foundation Trust and University of Birmingham, Birmingham, UK

**Keywords:** Malaria, Household air pollution, Children under 5 years, Low and middle-income country, Sub-Saharan Africa, Biomass

## Abstract

**Background:**

Smoke from solid biomass cooking is often stated to reduce household mosquito levels and, therefore, malarial transmission. However, household air pollution (HAP) from solid biomass cooking is estimated to be responsible for 1.67 times more deaths in children aged under 5 years compared to malaria globally. This cross-sectional study investigates the association between malaria and (i) cleaner fuel usage; (ii) wood compared to charcoal fuel; and, (iii) household cooking location, among children aged under 5 years in sub-Saharan Africa (SSA).

**Methods:**

Population-based data was obtained from Demographic and Health Surveys (DHS) for 85,263 children within 17 malaria-endemic sub-Saharan countries who were who were tested for malaria with a malarial rapid diagnostic test (RDT) or microscopy. To assess the independent association between malarial diagnosis (positive, negative), fuel type and cooking location (outdoor, indoor, attached to house), multivariable logistic regression was used, controlling for individual, household and contextual confounding factors.

**Results:**

Household use of solid biomass fuels and kerosene cooking fuels was associated with a 57% increase in the odds ratio of malarial infection after adjusting for confounding factors (RDT adjusted odds ratio (AOR):1.57 [1.30–1.91]; Microscopy AOR: 1.58 [1.23–2.04]) compared to cooking with cleaner fuels. A similar effect was observed when comparing wood to charcoal among solid biomass fuel users (RDT AOR: 1.77 [1.54–2.04]; Microscopy AOR: 1.21 [1.08–1.37]). Cooking in a separate building was associated with a 26% reduction in the odds of malarial infection (RDT AOR: 0.74 [0.66–0.83]; Microscopy AOR: 0.75 [0.67–0.84]) compared to indoor cooking; however no association was observed with outdoor cooking. Similar effects were observed within a sub-analysis of malarial mesoendemic areas only.

**Conclusion:**

Cleaner fuels and outdoor cooking practices associated with reduced smoke exposure were not observed to have an adverse effect upon malarial infection among children under 5 years in SSA. Further mixed-methods research will be required to further strengthen the evidence base concerning this risk paradigm and to support appropriate public health messaging in this context.

**Supplementary Information:**

The online version contains supplementary material available at 10.1186/s12936-022-04152-3.

## Background

Smoke arising from solid biomass cooking (wood, dung, charcoal, crop residue) is widely perceived to act as a mosquito repellent among communities [1–3], therefore protecting against mosquito-borne disease. However, solid biomass cooking produces health harming levels of household air pollution (HAP), estimated to be responsible for around 450,000 deaths in children aged under 5 years worldwide [4], compared to only 274,000 estimated deaths from malaria in 2019 [1]. This discordance in perceived compared to actual health risks associated with malarial transmission could impact upon uptake of structural interventions (e.g., cleaner fuel transition [LPG, electricity, biogas]) and behavioural changes intended to reduce harmful HAP exposure, notably among those living in endemic malarial regions.

Sub-Saharan Africa (SSA) has the highest malarial prevalence globally with 94% of cases and deaths, caused by predominantly by *Plasmodium falciparum* [5]. Identified risk factors for malarial infection include poor household construction [6–8] (e.g., open eaves), animals sleeping in the house [9] and presence of standing water near the house [10, 11]. The use of mosquito nets, household insecticidal spraying, and larval source management [12] have become common practice advocated in malarial prevention, driven in part by the World Health Organization’s (WHO) coordinated response [5]. Another, common preventive measure is use of mosquito repellent smoke from the burning of certain types of plant materials, such as *churai* in West Africa [2, 13].

There is little evidence supporting reduced mosquito infiltration [14, 15] or malarial transmission associated with solid biomass fuel cooking [2, 16]; although there is some evidence that solid biomass cooking reduces the risk of arboviruses in Guatemala [17]. Therefore, to better understand this disease risk paradigm, this study investigates the association of malarial acquisition among children aged under 5 years with regard to: (i) cleaner or solid biomass fuels and kerosene cooking; (ii) charcoal or wood fuel usage; and (iii) indoor and outdoor cooking, within households in 17 SSA countries using the population-based Demographic and Health Survey (DHS) data.

## Methods

### Data sources

**Fig. 1:**
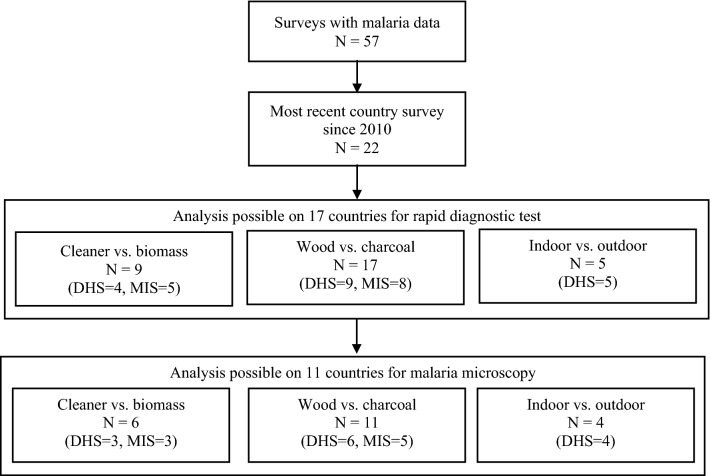
Flow diagram for included countries. *N* Number of countries

This cross-sectional study uses publicly available survey data for 17 malarial-endemic SSA countries with available malarial data (Fig. 1), obtained from the DHS program supported by the United States Agency for International Development (USAID) within the last 10 years (2010–2020). The DHS undertakes full surveys every 5 years, and intermediate Malaria Indicators Surveys (MIS) [18]; only some of the full DHS survey modules undertake malarial testing. For those DHS surveys including malaria modules, malarial testing is undertaken by trained fieldworkers on a sub-sample of eligible children aged 6–59 months using a malarial rapid diagnostic test (RDT) [18]. A two-stage stratified sampling technique was employed to obtain a representative population-based sample, with residential households randomly selected. Eligible households included those with an ever-married (married, widowed or divorced) woman aged between 15 and 49 years and resident the night before the survey. Ethical approval for data collection was gained from the relevant government authority [18], and authorization for data access was provided by the DHS.

Malarial endemicity was generated for each cluster by assessment of malarial prevalence obtained from the open source Malaria Atlas Project [19] within eligible countries, and defined as holoendemic (> 75%), hyperendemic (51–75%), mesoendemic (11–50%), hypoendemic (< 10%) [20]. Those data points that fell within hypoendemic areas were excluded from the analysis due to lower rate of malarial infection and testing. Malarial prevalence data were geocoded to the cluster geographic coordinates using the spatial analyst tool in ArcMAP 10.7 [21]; a method that has been previously used for this purpose [22].

As the wealth index provided by DHS contains cooking fuel as an indicator variable, a new modified wealth index was calculated in SPSS [23] using principal component analysis [24] to prevent circularity [8]. The index indicator variables included source of drinking water, house construction material, provision of a toilet facility and household assets, which varied by country (Additional file 1).

### Predictor and outcome variables

#### Proxies for household air pollution (HAP) exposure levels

Three analyses were undertaken (Table 1), undertaking comparisons by the main type of cooking fuel used and cooking location respectively: cleaner vs solid biomass fuels and kerosene fuels; charcoal vs wood fuels; outdoor vs indoor cooking (indoors, in a separate building).

**Table 1:** Analyses, sub-analyses and exploratory analyses undertaken with detail on categorisation of the exposure of interest

Analysis	Exposure of interest	Categories	Adjusted for	Sub-analysis	Exploratory analysis controlling for^‡^
Analysis 1	Biomass usage	• Cleaner (electricity, LPG, natural gas, biogas) • Solid biomass fuels and kerosene (kerosene, coal/lignite, charcoal, wood, straw/shrubs/grass, agricultural crop, animal dung)	• Child’s age, child’s gender, birth order, Child slept under slept under mosquito net last night, modified wealth index, number of household members, place of residence, malarial endemicity, season, cluster altitude and cooking location	• Urban areas only • Rural areas only • Mesoendemic areas only	• Household insecticidal spraying • Household smoking and cooking location
Analysis 2	Biomass fuel type*	• Charcoal • Wood	• Child’s age, child’s gender, birth order, Child slept under slept under mosquito net last night, modified wealth index, number of household members, place of residence, malarial endemicity, season, cluster altitude and cooking location	• Urban areas only • Rural areas only • Mesoendemic areas only	• Household insecticidal spraying • Household smoking and cooking location
Analysis 3	Cooking location^†^	• Outdoors • In a separate building • Indoors	• Child’s age, child’s gender, birth order, Child slept under slept under mosquito net last night, modified wealth index, number of household members, place of residence, malarial endemicity, season, cluster altitude and biomass cooking fuel type	• Urban areas only • Rural areas only • Mesoendemic areas only • Wood cooking only	• Household insecticidal spraying • Household smoking and cooking location

^*^Charcoal and wood are the most commonly used type of biomass fuel and are next to each other on fuel ladder, with charcoal being relatively less polluting

^†^Only Solid biomass fuels and kerosene (kerosene, coal/lignite, charcoal, wood, straw/shrubs/grass, agricultural crop, animal dung) were included in the analysis and included as a covariate

^‡^Countries excluded due to the variable being incomplete, high level of missing or low cell counts. For household insecticidal spraying excluded countries were: Burkina Faso 2017–2018, Cameron 2018, DRC 2013–2014, Malawi 2017, Mali 2018, Nigeria 2018, Tanzania 2017 and Togo 2017. For household smoking and cooking location excluded countries were: Burkina Faso 2017–2018, Ghana 2019, Liberia 2016, Malawi 2017, Mozambique 2018 and Sierra Leone 2016

#### Measure of malarial diagnosis

A malarial infection was determined by a positive RDT (n = 17 countries) and in some countries a subsequent blood smear test via microscopy taken at the point of interview (n = 11 countries), both of which were modelled as a binary (negative, positive) outcome variable, in separate analysis within this study. The RDT was undertaken using the SD BIOLINE Malaria Ag test, in all countries, which has estimated sensitivity of 99.7% and specificity of 99.5% [25]. Whereas, only certain countries collected blood samples which were collected with the parasites detected in the blood at time of survey using microscopy [18], with estimated sensitivity of 95.7% and specificity of 97.9% [26].

#### Explanatory variables

Covariates were included for the relevant contextual, household and individual factors identified as influencing both HAP exposure and malarial risk. Covariates were included in regression models as categorical variables other than household altitude, which was modelled as a continuous variable. Regional level variables were: malarial endemicity (mesoendemic, hyperendemic and holoendemic), season (dry, wet), rural or urban residence (rural, urban), cluster altitude (metres). Household level variables were: number of household members (≤ 6, > 6), household smoking (no, yes), modified wealth index (lowest, low, middle, high, highest), biomass cooking fuel type (where applicable; kerosene, coal/lignite, charcoal, wood, straw/shrubs/grass, agricultural crop, animal dung), household insecticide spraying within the last 12 months (no, yes) and dwelling construction (traditional, modern). Child variables were: age (< 1, 1, 2, 3, 4 years), birth order (first born, not first born), child’s sex (male, female), slept under mosquito net last night (no, yes—treated (ITN) net, yes—untreated net). The season variable is created using regional and country level information from the CIA fact book [27] and the World Bank climate change knowledge portal [28]. The household construction variable is a composite variable derived from the wall, roof and floor material. Firstly, each of the three materials were categorized into natural, rudimentary and finished construction material using the criteria outlined by Tusting et al. [8], followed by the creation of the household construction variable where modern household construction was define as wall, roof and floor being made of finished materials.

### Data analysis

Data preparation and analysis was undertaken in R studio [29]. Each variable was described within the combined dataset using number of cases (n), and percentage (%) and median and Interquartile range (IQR) for continuous variables. The level of missing data ranged from 0 to 48% of clinically relevant variables at a country level, which was imputed using the MICE package [30] with 50 iterations [31, 32]; to prevent bias from list-wise deletion [33]. To test the association between cooking practices and malarial infection, multivariable logistic regression using the survey package [34], was used to account for the complex sampling strategy; reporting adjusted odds ratios (AOR) and 95% confidence intervals (95% CI). The MIS survey did not contain information on cooking location and household smoking, therefore a sub-analysis was undertaken using countries where these variables were available for analysis. Sub-analyses were also undertaken for rural, urban, wood cooking fuel houses and mesoendemic areas. In addition, the analysis was repeated to include additional covariates among a sub-set of countries where additional variables of interest were available. This enabled investigation of the influence of (i) household cooking location; (ii) household smoking; and (iii) household insecticidal spraying, as some of the variables are missing from certain countries.

## Results

This study identified 85,263 children aged under 5 years children living in 17 participating countries (DHS = 9, MIS = 7) from 2011 to 2019, with a total of 74,461 RDT and 48,491 microscopy test results. Within the pooled full dataset, median child age was 3 years (IQR: 2–4). The proportion of girls ranged from 48.0% in Guinea (2012) to 51.0% in Cote d’Ivoire (2011–2012), with overall 49.4% in the pooled dataset (Table 2).

**Table 2:** Characteristics of included surveys

Country	Survey	N	Positive RDT (%)*	Positive microscopy (%)*	Child’s age (years) n (%)					Females (%)
					< 1	1	2	3	4	
Central Africa										
Cameroon 2018	DHS	4417	23.9	–	567 (12.8%)	873 (19.8%)	1056 (23.9%)	1002 (22.7%)	919 (20.8%)	48.7
DRC 2013–2014	DHS	6359	35.9	28.3	868 (13.6%)	1263 (19.9%)	1515 (23.8%)	1390 (21.9%)	1324 (20.8%)	50.1
East Africa										
Burundi 2016–2017	DHS	4309	47.4	33.4	604 (14.0%)	901 (20.9%)	935 (21.7%)	921 (21.4%)	948 (22.0%)	49.5
Malawi 2017	MIS	1929	41.3	–	229 (11.9%)	374 (19.4%)	438 (22.7%)	406 (21.1%)	480 (24.9%)	49.3
Mozambique 2018	MIS	384	45.4	–	507 (13.4%)	769 (20.3%)	944 (25.0%)	810 (21.4%)	753 (19.9%)	49.3
Tanzania 2017	MIS	5882	7.1	–	782 (13.3%)	1197 (20.3%)	1383 (23.5%)	1308 (22.2%)	1212 (20.6%)	49.7
Uganda 2018–2019	MIS	5282	21.0	11.3	631 (11.9%)	1011 (19.1%)	1281 (24.3%)	1228 (23.2%)	1131 (21.4%)	49.5
West Africa										
Benin 2017–2018	DHS	11,981	36.4	39.3	1747 (14.6%)	2390 (19.9%)	2705 (22.6%)	2699 (22.5%)	2440 (20.4%)	49.2
Burkina Faso 2017–2018	MIS	4839	20.8	17.1	645 (13.3%)	877 (18.1%)	1175 (24.3%)	1149 (23.7%)	992 (20.5%)	49.2
Cote d’Ivoire 2011–2012	DHS	3679	50	17.6	550 (14.9%)	749 (20.4%)	932 (25.3%)	808 (22.0%)	640 (17.4%)	51.0
Ghana 2019	MIS	2143	25.9	–	269 (12.6%)	407 (19.0%)	565 (26.4%)	457 (21.3%)	445 (20.8%)	49.1
Guinea 2021	DHS	3022	51.8	48.4	394 (13.0%)	580 (19.2%)	660 (21.8%)	729 (24.1%)	659 (21.8%)	48.0
Liberia 2016	DHS	3074	45.0	–	388 (12.6%)	581 (18.9%)	711 (23.1%)	712 (23.2%)	682 (22.2%)	49.1
Mali 2018	DHS	5159	26.4	–	664 (12.9%)	1117 (21.7%)	1224 (23.7%)	1126 (21.8%)	1028 (19.9%)	49.5
Nigeria 2018	DHS	9791	34.8	21.9	1335 (13.6%)	2017 (20.6%)	2273 (23.2%)	2153 (22.0%)	2013 (20.6%)	49.2
Sierra Leone 2016	MIS	6763	52.7	40.1	946 (14.0%)	1226 (18.1%)	1594 (23.6%)	1587 (23.5%)	1411 (20.9%)	50.0
Togo 2017	MIS	2850	44.3	28.8	401 (14.1%)	566 (19.8%)	666 (23.4%)	630 (22.1%)	588 (20.6%)	50.3

N: Number of child observations, DHS: Demographic and Health Survey, MIS: Malaria Indicators Survey, n: number of child observation with each category

^*^Percentage for positive results based on those children who received a conclusive result from malaria test

**Fig. 2:**
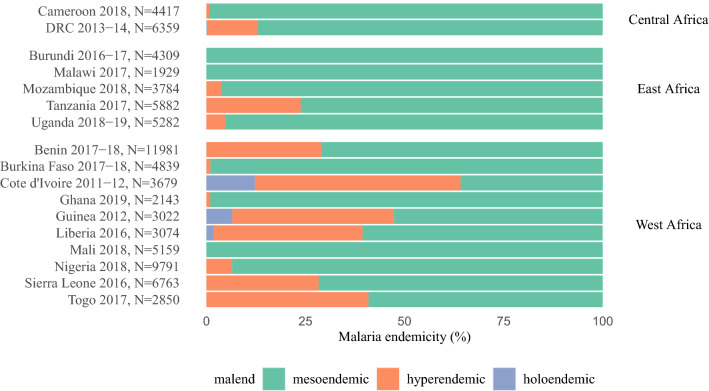
Malarial endemicity and prevalence among children under five for each country. *N* number of child observations, *PR* prevalence rate of positive RDT result

Malarial infection was positively identified by RDT among 34.6% of children in the combined dataset at the time of testing, with the highest point prevalence in Guinea 2012 (51.8%) and lowest in Tanzania 2017 (7.07%) (Table 3). However, where microscopy was undertaken malarial infection was identified in 28.2% of children, with the highest prevalence in Guinea 2012 (48.7%) and lowest in Uganda 2018–2019 (11.3%). Of the areas surveyed, most were in mesoendemic areas (Fig. 2), with holoendemicity in Cote d’Ivoire 2011–2012, DRC 2013–2014, Guinea 2012 and Liberia 2016. Of those children with a positive malarial RDT result, 1.3% resided in cleaner cooking households. Whereas, 35.2% in outdoor cooking households and 35.7% in a household where cooking was typically undertaken in a separate building (Table 3).

**Table 3:** Descriptive statistics for the combined dataset (N = 85,263)

	Malaria RDT result (N = 74,461)			Malaria Microscopy results (N = 48,491)		
	Negative N = 48,699 (65.4%)	Positive N = 25,761 (34.6%)	p value	Negative N = 34,802 (71.8%)	Positive N = 13,689 (28.2%)	p value
Proxies for HAP exposure levels						
Cooking fuel			< 0.001			< 0.001
Electricity	247 (0.5%)	47 (0.2%)		196 (0.6%)	22 (0.2%)	
LPG	2404 (4.9%)	295 (1.1%)		1287 (3.7%)	98 (0.7%)	
Natural gas	305 (0.6%)	9 (0.0%)		201 (0.6%)	7 (0.1%)	
Biogas	38 (0.1%)	8 (0.0%)		16 (0.0%)	7 (0.0%)	
Kerosene	1256 (2.6%)	220 (0.9%)		927 (2.7%)	107 (0.8%)	
Coal, lignite	155 (0.3%)	42 (0.2%)		103 (0.3%)	24 (0.2%)	
Charcoal	10,043 (20.7%)	2297 (8.9%)		6368 (18.3%)	1500 (11.0%)	
Wood	33,799 (69.5%)	22,397 (87.0%)		25,288 (72.8%)	11,602 (84.8%)	
Other biomass	370 (0.8%)	417 (1.6%)		358 (1.0%)	307 (2.2%)	
No food cooked in house	15 (0.0%)	8 (0.0%)		7 (0.0%)	2 (0.0%)	
Missing	68	22		50	13	
Cooking location			< 0.001			< 0.001
In the house	7108 (29.0%)	4129 (29.1%)		6326 (31.2%)	2830 (32.2%)	
In a separate building	9170 (37.5%)	5068 (35.7%)		6468 (31.9%)	2627 (29.9%)	
Outdoors	8196 (33.5%)	4994 (35.2%)		7482 (36.9%)	3321 (37.8%)	
Missing	24,226	11,571		14,526	4911	
Contextual and contextual variables						
Place of residence			< 0.001			< 0.001
Urban	17,582 (36.1%)	4683 (18.2%)		11,635 (33.4%)	2669 (19.5%)	
Season			< 0.001			< 0.001
Dry	25,169 (51.7%)	11,776 (45.7%)		20,750 (59.6%)	6583 (48.1%)	
Malarial endemicity			< 0.001			< 0.001
Mesoendemic	42,772 (87.8%)	19,018 (73.8%)		29,351 (84.3%)	9457 (69.1%)	
Hyperendemic	5729 (11.8%)	6286 (24.4%)		5116 (14.7%)	3971 (29.0%)	
Holoendemic	198 (0.4%)	457 (1.8%)		335 (1.0%)	261 (1.9%)	
Cluster altitude			< 0.001			< 0.001
Median IQR	294 (85, 596)	321 (156, 590)		322 (149, 764)	324 (149, 588)	
Household level variables						
Modified Wealth Index			< 0.001			< 0.001
Lowest	8669 (17.8%)	7714 (29.9%)		6633 (19.1%)	3976 (29.0%)	
Low	9618 (19.7%)	7306 (28.4%)		6925 (19.9%)	3722 (27.2%)	
Middle	9919 (20.4%)	5698 (22.1%)		6949 (20.0%)	2908 (21.2%)	
High	10,886 (22.4%)	3802 (14.8%)		7724 (22.2%)	2225 (16.3%)	
Highest	9608 (19.7%)	1241 (4.8%)		6569 (18.9%)	859 (6.3%)	
Household smoking			< 0.001			< 0.001
No	20,049 (81.6%)	10,852 (76.1%)		16,195 (79.5%)	6631 (75.1%)	
Missing	24,119	11,497		14,430	4860	
Number of household members			< 0.001			< 0.001
≤ 6	26,538 (54.6%)	13,007 (50.6%)		18,579 (53.5%)	6631 (48.5%)	
Missing	68	44		51	31	
Household insecticide spraying within last 12 months			< 0.001			< 0.001
No	18,189 (91.1%)	13,044 (94.9%)		17,582 (93.3%)	8527 (95.6%)	
Yes	1779 (8.9%)	703 (5.1%)		1260 (6.7%)	394 (4.4%)	
Missing	28,731	12,014		15,960	4768	
House construction			< 0.001			< 0.001
Traditional	28,361 (58.2%)	19,352 (75.1%)		20,902 (60.1%)	10,056 (73.5%)	
Modern	20,338 (41.8%)	6410 (24.9%)		13,900 (39.9%)	3634 (26.5%)	
Child level variables						
Child’s age (years)			< 0.001			< 0.001
< 1	7643 (15.7%)	2282 (8.9%)		5319 (15.3%)	1272 (9.3%)	
1	10,335 (21.2%)	4404 (17.1%)		7359 (21.1%)	2186 (16.0%)	
2	11,266 (23.1%)	6328 (24.6%)		8127 (23.4%)	3239 (23.7%)	
3	10,254 (21.1%)	6497 (25.2%)		7425 (21.3%)	3568 (26.1%)	
4	9201 (18.9%)	6251 (24.3%)		6572 (18.9%)	3424 (25.0%)	
Birth order			< 0.001			
First born	14,376 (33.7%)	5338 (24.5%)		9392 (30.8%)	2553 (22.1%)	
Missing	6102	3993		4306	2164	
Child’s gender			0.068			
Male	24,535 (50.4%)	13,112 (50.9%)		17,489 (50.3%)	6971 (50.9%)	
Child slept under slept under mosquito net last night			< 0.001			< 0.001
Did not sleep under a net	20,615 (42.3%)	12,078 (46.9%)		15,858 (45.6%)	6942 (50.7%)	
Only treated (ITN) nets	26,991 (55.4%)	13,204 (51.3%)		18,320 (52.6%)	6525 (47.7%)	
Only untreated nets	1093 (2.2%)	480 (1.9%)		624 (1.8%)	222 (1.6%)	

N: Number of observations; %: column percentage for categorical variables; IQR: interquartile range; ITN: insecticide-treated nets; RDT: rapid diagnostic test

### Analysis 1—Solid biomass fuel usage and risk of malarial infection

**Fig. 3:**
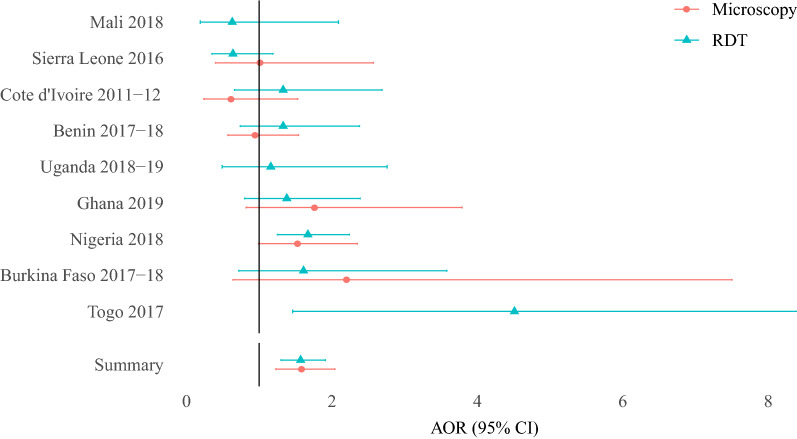
Adjusted odds ratio of malarial infection with biomass cooking compared to cleaner cooking. *AOR* adjusted odds ratio, *95% CI* 95% confidence interval, *N* Number of child observations. Table of unadjusted and adjusted results can be found in Additional file 2: Table S2.1

In pooled analyses, cooking with solid biomass fuels and kerosene fuels was observed to be independently associated with a 57% increase in the adjusted odds ratio for malarial infection, compared to cleaner cooking (electricity, LPG) (Fig. 3) (RDT AOR: 1.57 [1.30–1.914]; Microscopy AOR: 1.58 [1.23–2.04]) (Table 3). A 61% increase in adjusted odds ratio was also observed when investigating the effect of cooking location and household smoking with solid biomass fuels and kerosene compared to cleaner cooking fuels (RDT AOR: 1.61 [1.28–2.02]; Microscopy AOR: 1.61 [1.20–2.15]. The increased malarial infection adjusted odds ratio associated with solid biomass fuels and kerosene cooking remained in the stratified sub-analysis among rural locations (RDT AOR: 1.41 [1.02–1.95]; Microscopy AOR: 2.10 [1.34–3.32]), urban locations (RDT AOR: 1.58 [1.24–2.03] only) and mesoendemic regions (RDT AOR: 1.58 [1.28–1.95]; Microscopy AOR: 1.59 [1.21–2.08]) (Table 4).

**Table 4:** Odds ratio of malarial infection for each cooking practices for the combined dataset, exploratory and sub-analysis

Analysis	Outcome	Analysis 1 Biomass vs cleaner cooking				Analysis 2 Wood vs charcoal cooking				Analysis 3 Cooking location			
		Cooking fuel	AOR [95% CI]	*p* value	N	Cooking fuel	AOR [95% CI]	*p* value	N	Type of cooking location	AOR [95% CI]	*p* value	*N*
Combined dataset*	RDT	Cleaner	*Ref*.			Charcoal	*Ref*.			Indoor	*Ref*.		
		Biomass	**1.57 [1.30–1.91]**	**< 0.001**	43,759	Wood	**1.77 [1.54–2.04]**	**< 0.001**	73,072	In a separate building	**0.74 [0.66–0.83]**	**< 0.001**	23,754
										Outdoor	0.94 [0.83–1.05]	0.26	
	Microscopy	Cleaner	*Ref*.			Charcoal	*Ref*.			Indoor	*Ref*.		
		Biomass	**1.58 [1.23–2.04]**	**< 0.001**	30,007	Wood	**1.21 [1.08–1.37]**	**0.001**	46,206	In a separate building	**0.75 [0.67–0.84]**	**< 0.001**	21,383
										Outdoor	0.97 [0.86–1.09]	0.58	
Sub-analysis													
Rural areas	RDT	Cleaner	*Ref*.			Charcoal	*Ref*.			Indoor	*Ref*.		
		Biomass	**1.41 [1.02–1.95]**	**0.04**	31,100	Wood	**1.43 [1.21–1.70]**	**< 0.001**	54,473	In a separate building	**0.70 [0.62–0.80]**	**< 0.001**	16,988
										Outdoor	0.91 [0.79–1.04]	0.17	
	Microscopy	Cleaner	*Ref*.			Charcoal	*Ref*.			Indoor	*Ref*.		
		Biomass	**2.10 [1.34–3.32]**	**0.001**	20,290	Wood	1.09 [0.91–1.30]	0.36	34,693	In a separate building	**0.73 [0.64–0.84]**	**< 0.001**	15,193
										Outdoor	0.92 [0.80–1.07]	0.28	
Urban areas	RDT	Cleaner	*Ref*.			Charcoal	*Ref*.			Indoor	*Ref*.		
		Biomass	**1.58 [1.24–2.03]**	**< 0.001**	12,659	Wood	**2.23 [1.79–2.78]**	**< 0.001**	18,599	In a separate building	0.96 [0.78–1.19]	0.72	6766
										Outdoor	0.99 [0.81–1.21]	0.90	
	Microscopy	Cleaner	*Ref*.			Charcoal	*Ref*.			Indoor	*Ref*.		
		Biomass	1.30 [0.96–1.76]	0.09	9717	Wood	**1.40 [1.20–1.64]**	**< 0.001**	11,513	In a separate building	0.86 [0.69–1.07]	0.17	6190
										Outdoor	1.08 [0.90–1.31]	0.40	
Mesoendemic areas	RDT	Cleaner	*Ref*.			Charcoal	*Ref*.			Indoor	*Ref*.		
		Biomass	**1.58 [1.28–1.95]**	**< 0.001**	35,167	Wood	**1.77 [1.49–2.09]**	**< 0.001**	57,814	In a separate building	**0.73 [0.65–0.82]**	**< 0.001**	20,349
										Outdoor	0.92 [0.81–1.05]	0.22	
	Microscopy	Cleaner	*Ref*.			Charcoal	*Ref*.			Indoor	*Ref*.		
		Biomass	**1.59 [1.21–2.08]**	**0.001**	23,519	Wood	**1.26 [1.10–1.44]**	**0.001**	35,898	In a separate building	**0.73 [0.65–0.83]**	**< 0.001**	18,209
										Outdoor	0.94 [0.83–1.08]	0.37	
Wood only	RDT									Indoor	*Ref*.		
										In a separate building	**0.75 [0.67–0.85]**	**< 0.001**	19,406
										Outdoor	0.90 [0.79–1.02]	0.10	
	Microscopy									Indoor	*Ref*.		
										In a separate building	**0.77 [0.67–0.87]**	**< 0.001**	17,244
										Outdoor	0.94 [0.82–1.08]	0.36	
Exploratory analysis													
Controlling for household mosquito spraying^†^	RDT	Cleaner	*Ref*.			Charcoal	*Ref*.			Indoor	*Ref*.		
		Biomass	1.23 [0.94–1.61]	0.14	26,778	Wood	**1.94 [1.62–2.33]**	**< 0.001**	36,898	In a separate building	**0.85 [0.73–0.99]**	**0.03**	9951
										Outdoor	0.95 [0.81–1.10]	0.47	
	Microscopy	Cleaner	*Ref*.			Charcoal	*Ref*.			Indoor	*Ref*.		
		Biomass	1.07 [0.77–1.47]	0.69	18,102	Wood	**1.30 [1.13–1.49]**	**< 0.001**	27,115	In a separate building	**0.76 [0.65–0.88]**	**< 0.001**	9676
										Outdoor	0.92 [0.79–1.07]	0.29	

*AOR* Adjusted odds ratio, *95% CI* 95% confidence interval, *N* Number of observations, *RDT* Rapid diagnostic test, *Ref* Reference group. Results in bold are statistically significant. Unadjusted results are in Additional file 3: Table S3.1

^*^Controlled for: Child’s age, child’s gender, birth order, Child slept under slept under mosquito net last night, modified wealth index, number of household members, place of residence, malarial endemicity, season and cluster altitude

^†^Burkina Faso 2017–2018, Cameron 2018, DRC 2013–2014, Malawi 2017, Mali 2018, Nigeria 2018, Tanzania 2017 and Togo 2017 were excluded due to the household mosquito spraying variable being incomplete, high level of missing or low cell counts

### Analysis 2—Biomass fuel type and risk of malarial infection

**Fig. 4:**
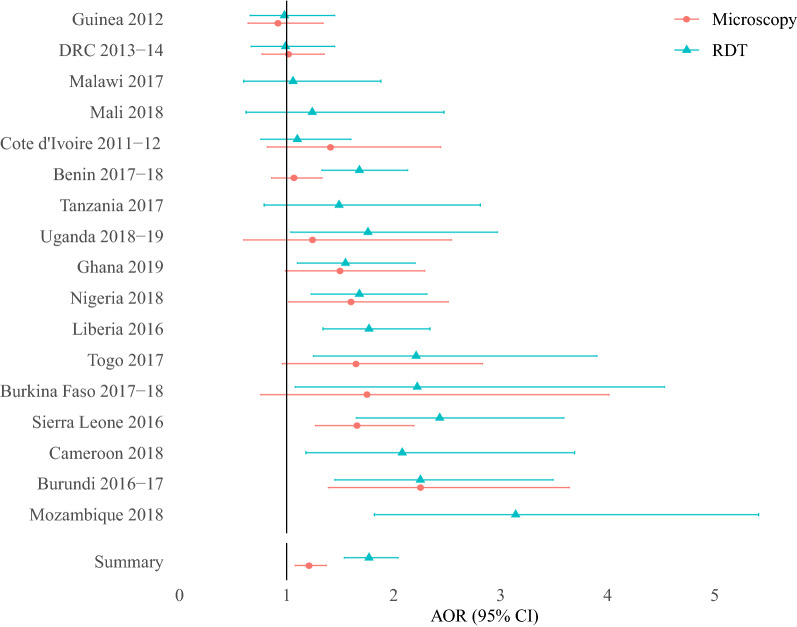
Adjusted odds ratio of malarial infection with wood cooking compared to charcoal cooking. *AOR* Adjusted odds ratio, *95% CI* 95% confidence interval, *N* Number of child observations. Table of unadjusted and adjusted results can be found in Additional file 2: Table S2.2

Among biomass fuel households only, use of wood compared to charcoal fuel was associated with an increased adjusted odds ratio of malarial infection (RDT AOR: 1.77 [1.54–2.04]; Microscopy AOR: 1.21 [1.08–1.37]) (Fig. 4), with a similar effect being observed in the exploratory analysis controlling for cooking location and household smoking (RDT AOR: 1.26 [1.10–1.46] only) and in mesoendemic areas (RDT AOR: 1.77 [1.49–2.09]; Microscopy AOR: 1.26; [1.10–1.44]) (Table 4). In the stratified sub-analysis it was observed that urban areas had a greater adjusted odds ratio of malarial infection associated with wood compared to charcoal cooking (RDT AOR: 2.25 [1.79–2.78]), in comparison to rural areas (RDT AOR: 1.43 [1.21–1.70]).

### Analysis 3—Household cooking location and risk of malarial infection

**Fig. 5:**
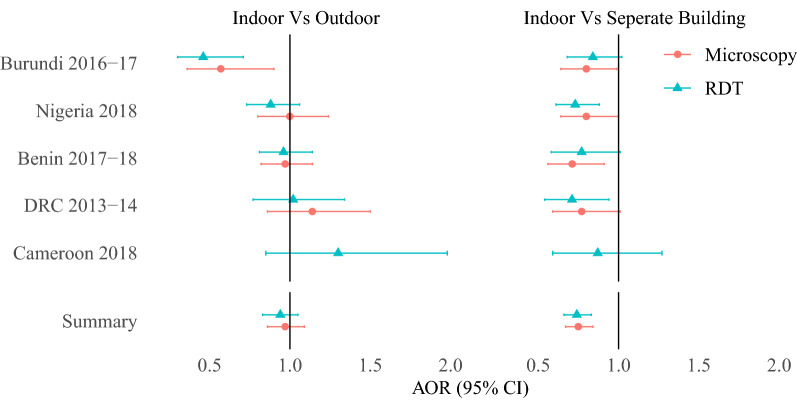
Adjusted odds ratio of malarial infection with cooking location (outdoor, in a separate building) compared to indoors. *AOR* Adjusted odds ratio, *95% CI* 95% confidence interval, *N* Number of child observations. Table of unadjusted and adjusted results can be found in Additional file 2: Table S2.3

No significant association was observed between household cooking location and malaria adjusted odds ratio (RDT AOR: 0.94 [0.83–1.05]; Microscopy AOR: 0.97 [95% CI 0.83–1.05]) (Fig. 5). In comparison, cooking in a separate building was associated with a reduced adjusted odds ratio of malarial infection by 74% compared to indoor cooking (Fig. 5) (RDT AOR: 0.74 [0.66–0.83]; Microscopy AOR: 0.75 [0.67–0.84]). The same reduced malarial infection adjusted odds ratio associated with cooking in a separate building was observed in stratified sub-analyses for wood cooking (RDT AOR: 0.75 [0.67–0.85]; Microscopy AOR: 0.77 [0.67–0.87]), rural (RDT AOR: 0.70 [0.62–0.80]; Microscopy AOR: 0.73 [0.64–0.84]) and mesoendemic areas (RDT AOR: 0.73 [0.65–0.82]; Microscopy AOR: 0.74 [0.65–0.83) only (Table 4).

## Discussion

This large exploratory study of over 85,000 children aged under 5 years living in 17 malaria-endemic SSA found no evidence to suggest that use of cleaner fuels (e.g., LPG, electricity, biogas), charcoal vs wood, or outdoor cooking location are associated with an increased risk of malarial infection. Indeed, the findings suggest that solid biomass fuel usage may be associated with a higher incidence of malarial infection among children in SSA. There are a number of factors that may account for the increase in infections, such as the longer cooking times and thus of carbon dioxide production [35], a major mosquito attractant [36], found with solid biomass fuel cooking [37]. Additionally, the use of solid biomass fuels, particularly wood, crop residue and dung, require women, to typically collect fallen or harvest branches from woods and forests where mosquitoes commonly reside, often taking children under 5 years on their backs, thereby increasing risk of mosquito bites.

It is highly likely that risk of within household acquisition of malaria is also influenced by socioeconomic factors such as household construction characteristics (eaves space, wall type) and living conditions [8, 38–41] which are not fully captured in the DHS composite wealth index. It is also recognized that use of cleaner domestic energy sources, cooking in a separate building and selection of biomass cooking fuel type may reflect socio-economic determinants, also related to malarial microepidemiology at the household level [42, 43]. The child’s age is also a key factor in malarial infection risk, with an observed increased risk with increasing age, potentially reflecting behavioural, nutritional or exposure differences. In terms of modifiable factors for malarial infection prevention and control, there is strong evidence supporting the sustained use of ITN bed nets, larval source management and household insecticide spraying [12]; of which only ITN bed nets could be controlled for in the main analyses. The importance of household insecticide spraying can be seen in the subsidiary analysis undertaken among countries for which this information was available, identifying that there was no association with type of biomass fuel and malarial infection risk (RDT: AOR 1.23 [0.94–1.61]; Microscopy AOR: 1.07 [0.77–1.47]; Table 4); however, this sub-analysis is likely to be underpowered and should be interpreted with caution.

The analyses presented also did not explore broader contextual factors associated with household or village level clustering of malarial transmission, including position of households in relation to mosquito sites and local attitudes to malarial treatment which are recognized to influence local variations in malarial prevalence [44]. The DHS dataset did not contain information on cooking practices such as timing or duration, both of which influence the amount of smoke produced and therefore HAP exposure, and may also generate higher localized levels of indoor CO_2_ [35] thereby attracting mosquitoes into the home [36]. In addition, season could only be accounted for at country or broader regional level, which does not take into account microclimates, in addition, the DHS is normally undertaken in the dry season and the MIS in the wet season when the malarial transmission risk is increased [18]. HAP interventions should be developed to include activities which communicate that cooking practices which produce less smoke do not increase risk of malaria transmission to residents. It is also important to reinforce health protection advice regarding evidence-based measures for mosquito control. Further qualitative and quantitative research is merited, for a detailed investigation of the relationships between cooking location, fuel choice and risk of malarial acquisition, considering a wider range of transmission risk factors at a local level.

The rural–urban differences in cooking activity patterns, which can be most clearly noted within the differences observed in distribution between fuel types, is likely to reflect relative economic development, improved access to cleaner fuel sources in urban areas and reduced potential for cohabitation with livestock [45]. However, the rural–urban divide was not as distinct within the cleaner fuel or cooking location sub-analysis, indicating that other contextual and compositional factors exist which may influence malarial infection risk (e.g., nutrition). Although season, malarial endemicity and altitude were captured as confounding factors within our analyses, information was not available for other contextual factors of relevance to malarial infection risk, such as temperature [46].

Additionally, although the cooking practices are reported at the time of interview, this survey question does not take into consideration longer-term trends which may vary on a seasonal basis. Further prospective research is required to better understand environmental influences upon malarial microepidemiology including objective pollutant exposure assessment, capture of household design characteristics, lifestyle and time-activity factors to assess relationships with mosquito breeding conditions, malarial parasitaemia and outcomes among adults and children.

## Conclusion

This large-scale observational study suggests that use of cleaner fuels and outdoor cooking practices typically associated with lower levels of household smoke, were not associated with an increased malarial acquisition risk among children living in SSA. Further mixed-methods research is required to better understand the relationships between cooking practices, cooking fuel emissions, mosquito activity and risk of malarial acquisition at household and community levels in this world region.

## Supplementary Information

**Additional file 1: Table S1.1.** Predictors included with the PCA analysis for the modified wealth index by country.

**Additional file 2: Table S2.1.** Unadjusted and adjusted odds ratio of malarial infection with solid biomass fuels and kerosene cooking compared to cleaner cooking—Analysis 1. **Table S2.2.** Unadjusted and adjusted odds ratio of malarial infection with wood cooking compared to charcoal cooking—Analysis 2.** Table S2.3.** Unadjusted and adjusted odds ratio of malarial infection with cooking location (outdoor, in a separate building) compared to indoors—Analysis 3.

**Additional file 3: Table S3.1.** Unadjusted odds ratio of malarial infection for each cooking practices for the combined dataset, exploratory and sub-analysis.

## Data Availability

The data that support the findings of this study freely and publicly available from https://dhsprogram.com/data/ and https://malariaatlas.org/.

## Abbreviations

AOR: Adjusted odds ratio
DHS: Demographic and Health Survey
DRC: Democratic Republic of Congo
HAP: Household air pollution
ITN: Insecticide-treated net
LPG: Liquefied petroleum gas
MICE: Multiple imputation by chained equations
MIS: Malaria indicator Survey
RDT: Rapid diagnostic test
SSA: Sub-Saharan Africa
USAID: United States Agency for International Development
95% CI: 95% Confidence interval
