# Supplementary material for: Cooking outdoors or with cleaner fuels does not increase malarial risk in children under 5 years: a cross-sectional study of 17 sub-Saharan African countries
Source: Malar J. 2022 Apr 27;21:133. doi: 10.1186/s12936-022-04152-3 (PMC9044678; doi:10.1186/s12936-022-04152-3)
Supplement: Supplementary file 1 — Additional file 1: Table S1.1. Predictors included with the PCA analysis for the modified wealth index by country. [file 12936_2022_4152_MOESM1_ESM.docx]

**Additional File 1**

**Table S1.1: Predictors included with the PCA analysis for the modified wealth index by country**

| Country | Source of drinking water | Toilet facility | House construction | | | Assets | | | | | | | | | | | | | | | | | | | Dwelling window material | Lighting fuel | Refuse collection | Own or rent house |
| --- | --- | --- | --- | --- | --- | --- | --- | --- | --- | --- | --- | --- | --- | --- | --- | --- | --- | --- | --- | --- | --- | --- | --- | --- | --- | --- | --- | --- |
|  |  |  | Wall material | Roof material | Floor material | Electricity | Radio | television | refrigerator | Watch | Bicycle | Motorcycle or scooter | Animal-drawn cart | Car or Truck | Boat with a motor | Bank account | Mobile telephone | Computer | Non-motorised boat/canoe | Tractor | Plough | Household furniture* | Household electronics* | Other assets* |  |  |  |  |
| Benin 2017-18 |  |  |  |  |  |  |  |  |  |  |  |  |  |  |  |  |  |  |  |  |  |  |  |  |  |  |  |  |
| Burkina Faso 2017-18 |  |  |  |  |  |  |  |  |  |  |  |  |  |  |  |  |  |  |  |  |  |  |  |  |  |  |  |  |
| Burundi 2016-17 |  |  |  |  |  |  |  |  |  |  |  |  |  |  |  |  |  |  |  |  |  |  |  |  |  |  |  |  |
| Cameroon 2018 |  |  |  |  |  |  |  |  |  |  |  |  |  |  |  |  |  |  |  |  |  |  |  |  |  |  |  |  |
| Côte d'Ivoire 2011-12 |  |  |  |  |  |  |  |  |  |  |  |  |  |  |  |  |  |  |  |  |  |  |  |  |  |  |  |  |
| DRC 2014-15 |  |  |  |  |  |  |  |  |  |  |  |  |  |  |  |  |  |  |  |  |  |  |  |  |  |  |  |  |
| Ghana 2019 |  |  |  |  |  |  |  |  |  |  |  |  |  |  |  |  |  |  |  |  |  |  |  |  |  |  |  |  |
| Guinea 2012 |  |  |  |  |  |  |  |  |  |  |  |  |  |  |  |  |  |  |  |  |  |  |  |  |  |  |  |  |
| Liberia 2016 |  |  |  |  |  |  |  |  |  |  |  |  |  |  |  |  |  |  |  |  |  |  |  |  |  |  |  |  |
| Malawi 2017 |  |  |  |  |  |  |  |  |  |  |  |  |  |  |  |  |  |  |  |  |  |  |  |  |  |  |  |  |
| Mali 2018 |  |  |  |  |  |  |  |  |  |  |  |  |  |  |  |  |  |  |  |  |  |  |  |  |  |  |  |  |
| Mozambique 2018 |  |  |  |  |  |  |  |  |  |  |  |  |  |  |  |  |  |  |  |  |  |  |  |  |  |  |  |  |
| Nigeria 2018 |  |  |  |  |  |  |  |  |  |  |  |  |  |  |  |  |  |  |  |  |  |  |  |  |  |  |  |  |
| Sierra Leone 2016 |  |  |  |  |  |  |  |  |  |  |  |  |  |  |  |  |  |  |  |  |  |  |  |  |  |  |  |  |
| Tanzania 2017 |  |  |  |  |  |  |  |  |  |  |  |  |  |  |  |  |  |  |  |  |  |  |  |  |  |  |  |  |
| Togo 2017 |  |  |  |  |  |  |  |  |  |  |  |  |  |  |  |  |  |  |  |  |  |  |  |  |  |  |  |  |
| Uganda 2018-19 |  |  |  |  |  |  |  |  |  |  |  |  |  |  |  |  |  |  |  |  |  |  |  |  |  |  |  |  |
| *Household furniture (e.g., table, chairs, wardrobe, bed, mattress, lamps, clock) | | | | | | | | | | | | | | | | | | | | | | | | | | | | |
| *Household electronics (e.g., washing machine, DVD player, internet, modem/router, satellite, laptop, Generator, music system, sewing machine, fan, air conditioning, solar panel, water pump, battery, iron, TV5 antenna, Cable subscription, camera, blender, microwave) | | | | | | | | | | | | | | | | | | | | | | | | | | | | |
| *Other assets (e.g., Grain mill, hammer mill, Rickshaw/Chingchi/Tuk tuk/Htawlargyi/Keke Napep/Bagag, bank account with another institution, credit union, beneficiary of Pantawid Pamilyan Pilipino Program (4Ps), canoe with motor, banana boat, thresher, bedroom available for sleep, floor area of house) | | | | | | | | | | | | | | | | | | | | | | | | | | | | |
