# Supplementary material for: Cooking outdoors or with cleaner fuels does not increase malarial risk in children under 5 years: a cross-sectional study of 17 sub-Saharan African countries
Source: Malar J. 2022 Apr 27;21:133. doi: 10.1186/s12936-022-04152-3 (PMC9044678; doi:10.1186/s12936-022-04152-3)
Supplement: Supplementary file 2 — Additional file 2: Table S2.1. Unadjusted and adjusted odds ratio of malarial infection with solid biomass fuels and kerosene cooking compared to cleaner cooking—Analysis 1. Table S2.2. Unadjusted and adjusted odds ratio of malarial infection with wood cooking compared to charcoal cooking—Analysis 2. Table S2.3. Unadjusted and adjusted odds ratio of malarial infection with cooking location (outdoor, in a separate building) compared to indoors—Analysis 3. [file 12936_2022_4152_MOESM2_ESM.docx]

**Additional file 2**

| **Table S2.1: Unadjusted and adjusted odds ratio of malarial infection with solid biomass fuels and kerosene cooking compared to cleaner cooking – Analysis 1** | | | | | | |
| --- | --- | --- | --- | --- | --- | --- |
| Country | Outcome | UOR [95% CI] | *p* value | AOR [95% CI] | *p* value | N |
| Benin 2017-18 | RDT | **5.13[2.79-9.43]** | **<0.001** | 1.33[0.74-2.38] | 0.35 | 6118 |
|  | Microscopy | **2.02[1.28-3.20]** | **0.003** | 0.94[0.57-1.54] | 0.80 | 5829 |
| Burkina Faso 2017-18 | RDT | **4.19[2.00-8.77]** | **<0.001** | 1.61[0.72-3.58] | 0.25 | 4845 |
|  | Microscopy | **5.49[1.69-17.86]** | **0.005** | 2.20[0.64-7.50] | 0.21 | 4840 |
| Côte d'Ivoire 2011-12 | RDT | **7.31[4.63-11.56]** | **<0.001** | 1.33[0.66-2.69] | 0.43 | 3383 |
|  | Microscopy | **4.45[2.25-7.85]** | **<0.001** | 0.61[0.24-1.53] | 0.29 | 3248 |
| Ghana 2019 | RDT | **3.54[2.11-5.94]** | **<0.001** | 1.38[0.80-2.39] | 0.25 | 2354 |
|  | Microscopy | **3.81[1.90-7.64]** | **<0.001** | 1.76[0.82-3.79] | 0.15 | 2356 |
| Mali 2018 | RDT | 1.04[0.44-2.50] | 0.92 | 0.63[0.19-2.09] | 0.45 | 2322 |
| Nigeria 2018 | RDT | **5.10[3.90-6.67]** | **<0.001** | **1.67[1.25-2.24]** | **0.001** | 9697 |
|  | Microscopy | **5.20[3.52-7.70]** | **<0.001** | 1.53[0.99-2.35] | 0.05 | 7065 |
| Sierra Leone 2016 | RDT | 0.88[0.30-2.57] | 0.81 | 0.64[0.35-1.19] | 0.16 | 6659 |
|  | Microscopy | 1.03[0.44-2.40] | 0.95 | 1.01[0.40-2.57] | 0.98 | 6669 |
| Togo 2017 | RDT | **27.29[9.83-75.76]** | **<0.001** | **4.51[1.46-13.9]** | **0.01** | 2905 |
| Uganda 2018-19 | RDT | 1.30[0.60-2.80] | 0.50 | 1.16[0.49-2.76] | 0.73 | 5476 |
| Abbreviation: UOR = Unadjusted Odds Ratio, AOR = Adjusted Odds Ratio, 95% CI = 95% confidence interval, N= Number of observations, RDT = Rapid diagnostic test. Results in bold are statistically significant.  The reported odds ratios are from solid biomass fuels and kerosene cooking with the reference group being cleaner cooking | | | | | | |

| **Table S2.2: Unadjusted and adjusted odds ratio of malarial infection with wood cooking compared to charcoal cooking –Analysis 2** | | | | | | |
| --- | --- | --- | --- | --- | --- | --- |
| Country | Outcome | UOR [95% CI] | *p* value | AOR [95% CI] | *p* value | N |
| Benin 2017-18 | RDT | **4.14[3.35-5.12]** | **<0.001** | **1.68[1.33-2.13]** | **<0.001** | 5670 |
|  | Microscopy | **1.95[1.60-2.38]** | **<0.001** | 1.07[0.86-1.33] | 0.54 | 5829 |
| Burkina Faso 2017-18 | RDT | **3.70[1.80-7.60]** | **<0.001** | 2.22[1.08-4.53] | 0.03 | 4697 |
|  | Microscopy | **3.04[1.31-7.04]** | **0.01** | 1.75[0.76-4.01] | 0.19 | 4840 |
| Burundi 2016-17 | RDT | **4.82[3.14-7.39]** | **<0.001** | **2.25[1.45-3.49]** | **<0.001** | 3739 |
|  | Microscopy | **4.62[2.88-7.40]** | **<0.001** | **2.25[1.39-3.64]** | **0.001** | 3743 |
| Cameroon 2018 | RDT | **2.73[1.68-4.44]** | **<0.001** | **2.08[1.18-3.69]** | **0.01** | 3367 |
| Côte d'Ivoire 2011-12 | RDT | **2.97[2.10-4.19]** | **<0.001** | 1.10[0.76-1.60] | 0.61 | 3105 |
|  | Microscopy | **3.82[2.30-6.34]** | **<0.001** | 1.41[0.82-2.44] | 0.22 | 2980 |
| DRC 2014-15 | RDT | 1.36[1.00-1.86] | 0.05 | 0.99[0.67-1.45] | 0.95 | 6181 |
|  | Microscopy | 1.19[0.92-1.54] | 0.20 | 1.02[0.77-1.35] | 0.91 | 6171 |
| Ghana 2019 | RDT | **2.30[1.65-3.22]** | **<0.001** | **1.55[1.10-2.20]** | **0.01** | 2098 |
|  | Microscopy | **2.17[1.56-3.02]** | **<0.001** | 1.50[0.99-2.29] | 0.06 | 2101 |
| Guinea 2012 | RDT | **2.26[1.64-3.11]** | **<0.001** | 0.98[0.66-1.45] | 0.93 | 2948 |
|  | Microscopy | **1.79[1.31-2.43]** | **<0.001** | 0.92[0.64-1.34] | 0.68 | 2949 |
| Liberia 2016 | RDT | **5.63[4.25-7.46]** | **<0.001** | **1.77[1.34-2.34]** | **<0.001** | 2785 |
| Malawi 2017 | RDT | **2.24[1.36-3.68]** | **0.002** | 1.06[0.60-1.88] | 0.84 | 1358 |
| Mali 2018 | RDT | 2.20[0.98-4.92] | 0.05 | 1.24[0.62-2.47] | 0.54 | 2244 |
| Mozambique 2018 | RDT | **6.95[4.08-11.84]** | **<0.001** | **3.14[1.82-5.41]** | **<0.001** | 3141 |
| Nigeria 2018 | RDT | **2.56[1.90-3.46]** | **<0.001** | **1.68[1.23-2.31]** | **0.001** | 7487 |
|  | Microscopy | **2.70[1.82-3.99]** | **<0.001** | **1.60[1.02-2.51]** | **0.04** | 5423 |
| Sierra Leone 2016 | RDT | **6.23[4.66-8.34]** | **<0.001** | **2.43[1.65-3.59]** | **<0.001** | 6612 |
|  | Microscopy | **3.67[2.92-4.61]** | **<0.001** | **1.66[1.27-2.19]** | **<0.001** | 6622 |
| Tanzania 2017 | RDT | **5.42[3.05-9.62]** | **<0.001** | 1.49[0.79-2.81] | 0.22 | 4903 |
| Togo 2017 | RDT | **6.01[4.24-8.51]** | **<0.001** | **2.21[1.25-3.90]** | **0.01** | 2791 |
|  | Microscopy | **4.18[2.90-6.02]** | **<0.001** | 1.65[0.96-2.83] | 0.07 | 2800 |
| Uganda 2018-19 | RDT | **3.09[1.73-5.51]** | **<0.001** | **1.76[1.04-2.97]** | **0.03** | 5426 |
|  | Microscopy | **2.63[1.18-5.85]** | **0.019** | 1.24[0.60-2.54] | 0.57 | 5421 |
| Abbreviation: UOR = Unadjusted Odds Ratio, AOR = Adjusted Odds Ratio, 95% CI = 95% confidence interval, N= Number of observations, RDT = Rapid diagnostic test. Results in bold are statistically significant.  The reported odds ratios are from wood cooking with the reference group being charcoal cooking | | | | | | |

| **Table S2.3: Unadjusted and adjusted odds ratio of malarial infection with cooking location (outdoor, in a separate building) compared to indoors – Analysis 3** | | | | | | | |
| --- | --- | --- | --- | --- | --- | --- | --- |
| Country | Outcome | Type of cooking location | UOR [95% CI] | *p* value | AOR [95% CI] | *p* value | N |
| Benin 2017-18 | RDT | In a separate building | **0.74[0.58-0.94]** | **0.02** | 0.77[0.58-1.01] | 0.06 | 5932 |
|  |  | Outdoor | 1.15[0.97-1.36] | 0.11 | 0.96[0.81-1.14] | 0.63 |  |
|  | Microscopy | In a separate building | **0.69[0.54-0.87]** | **0.002** | **0.71[0.56-0.91]** | **0.01** | 5653 |
|  |  | Outdoor | 1.07[0.91-1.25] | 0.43 | 0.97[0.82-1.14] | 0.67 |  |
| Burundi 2016-17 | RDT | In a separate building | **0.52[0.44-0.62]** | **<0.001** | 0.84[0.68-1.02] | 0.08 | 4019 |
|  |  | Outdoor | **0.30[0.20-0.44]** | **<0.001** | **0.46[0.30-0.71]** | **0.001** |  |
|  | Microscopy | In a separate building | **0.55[0.46-0.66]** | **<0.001** | **0.80[0.64-0.99]** | **0.04** | 4023 |
|  |  | Outdoor | **0.36[0.24-0.56]** | **<0.001** | **0.57[0.36-0.90]** | **0.02** |  |
| Cameroon 2018 | RDT | In a separate building | 0.94[0.64-1.37] | 0.73 | 0.87[0.59-1.27] | 0.46 | 3385 |
|  |  | Outdoor | 1.16[0.76-1.76] | 0.49 | 1.30[0.85-1.98] | 0.23 |  |
| DRC 2014-15 | RDT | In a separate building | 0.78[0.59-1.02] | 0.07 | **0.71[0.54-0.94]** | **0.02** | 6214 |
|  |  | Outdoor | 0.83[0.64-1.07] | 0.15 | 1.02[0.77-1.34] | 0.89 |  |
|  | Microscopy | In a separate building | 0.80[0.62-1.03] | 0.09 | 0.77[0.59-1.01] | 0.06 | 6204 |
|  |  | Outdoor | 0.88[0.69-1.13] | 0.33 | 1.14[0.86-1.50] | 0.36 |  |
| Nigeria 2018 | RDT | In a separate building | **0.69[0.57-0.73]** | **<0.001** | **0.78[0.61-0.88]** | **0.001** | 7589 |
|  |  | Outdoor | 0.88[0.74-1.06] | 0.17 | 0.88[0.73-1.06] | 0.18 |  |
|  | Microscopy | In a separate building | **0.71[0.57-0.88]** | **0.002** | 1.00[0.80-1.24] | 0.97 | 5503 |
|  |  | Outdoor | 0.94[0.76-1.16] | 0.57 | 0.80[0.64-1.00] | 0.05 |  |
| Abbreviation: UOR = Unadjusted Odds Ratio, AOR = Adjusted Odds Ratio, 95% CI = 95% confidence interval, N= Number of observations, RDT = Rapid diagnostic test. Results in bold are statistically significant.  The reference group is indoor cooking for all reported odds ratios. | | | | | | | |
