# Supplementary material for: Cooking outdoors or with cleaner fuels does not increase malarial risk in children under 5 years: a cross-sectional study of 17 sub-Saharan African countries
Source: Malar J. 2022 Apr 27;21:133. doi: 10.1186/s12936-022-04152-3 (PMC9044678; doi:10.1186/s12936-022-04152-3)
Supplement: Supplementary file 3 — Additional file 3: Table S3.1. Unadjusted odds ratio of malarial infection for each cooking practices for the combined dataset, exploratory and sub-analysis. [file 12936_2022_4152_MOESM3_ESM.docx]

**Additional file 3**

**Table S3.1: Unadjusted odds ratio of malarial infection for each cooking practices for the combined dataset, exploratory and sub-analysis**

| **Analysis** | **Outcome** | **Analysis 1**  **Biomass vs cleaner cooking** | | | | **Analysis 2**  **Wood vs charcoal cooking** | | | | **Analysis 3**  **Cooking location** | | | |
| --- | --- | --- | --- | --- | --- | --- | --- | --- | --- | --- | --- | --- | --- |
|  |  | Cooking fuel | AOR [95% CI] | *p* value | N | Cooking fuel | AOR [95% CI] | *p* value | N | Type of cooking location | AOR [95% CI] | *p* value | *N* |
| Combined dataset* | RDT | Cleaner | *Ref.* |  |  | Charcoal | *Ref.* |  |  | Indoor | *Ref.* |  |  |
|  |  | Biomass | **4.75[3.95-5.71]** | **<0.001** | 43759 | Wood | **3.06[2.71-3.44]** | **<0.001** | 73072 | In a separate building | **0.71[0.64-0.88]** | **<0.001** | 23754 |
|  |  |  |  |  |  |  |  |  |  | Outdoor | **0.80[0.72-0.79]** | **<0.001** |  |
|  | Microscopy | Cleaner | *Ref.* |  |  | Charcoal | *Ref.* |  |  | Indoor | *Ref.* |  |  |
|  |  | Biomass | **5.13[4.07-6.48]** | **<0.001** | 30007 | Wood | **1.94[1.73-2.18]** | **<0.001** | 46206 | In a separate building | **0.65[0.58-0.72]** | **<0.001** | 21383 |
|  |  |  |  |  |  |  |  |  |  | Outdoor | **0.89[0.80-1.00]** | **0.50** |  |
| **Sub-analysis** | | | | | | | | | | | | | |
| Rural areas | RDT | Cleaner | *Ref.* |  |  | Charcoal | *Ref.* |  |  | Indoor | *Ref.* |  |  |
|  |  | Biomass | **2.39[1.72-3.31]** | **<0.001** | 31100 | Wood | **1.73[1.46-2.05]** | **<0.001** | 54473 | In a separate building | **0.63[0.56-0.71]** | **<0.001** | 16988 |
|  |  |  |  |  |  |  |  |  |  | Outdoor | **0.78[0.68-0.89]** | **<0.001** |  |
|  | Microscopy | Cleaner | *Ref.* |  |  | Charcoal | *Ref.* |  |  | Indoor | *Ref.* |  |  |
|  |  | Biomass | **3.84[2.42-6.12]** | **<0.001** | 20290 | Wood | 1.26[1.05-1.52] | 0.015 | 34693 | In a separate building | **0.62[0.54-0.70]** | **<0.001** | 15193 |
|  |  |  |  |  |  |  |  |  |  | Outdoor | 0.89[0.77-1.02] | 0.10 |  |
| Urban areas | RDT | Cleaner | *Ref.* |  |  | Charcoal | *Ref.* |  |  | Indoor | *Ref.* |  |  |
|  |  | Biomass | **3.49[2.74-4.45]** | **<0.001** | 12659 | Wood | **2.82[2.33-3.43]** | **<0.001** | 18599 | In a separate building | 1.02[0.83-1.27] | 0.83 | 6766 |
|  |  |  |  |  |  |  |  |  |  | Outdoor | 1.19[0.99-1.41] | 0.61 |  |
|  | Microscopy | Cleaner | *Ref.* |  |  | Charcoal | *Ref.* |  |  | Indoor | *Ref.* |  |  |
|  |  | Biomass | **3.57[2.70-4.72]** | **<0.001** | 9717 | Wood | **1.77[1.52-2.06]** | **<0.001** | 11513 | In a separate building | **0.75[0.60-0.94]** | **0.01** | 6190 |
|  |  |  |  |  |  |  |  |  |  | Outdoor | 1.13[0.94-1.36] | 0.20 |  |
| Mesoendemic areas | RDT | Cleaner | *Ref.* |  |  | Charcoal | *Ref.* |  |  | Indoor | *Ref.* |  |  |
|  |  | Biomass | **4.26[3.50-5.19]** | **<0.001** | 35167 | Wood | **2.78[2.43-3.17]** | **<0.001** | 57814 | In a separate building | **0.72[0.64-0.80]** | **<0.001** | 20349 |
|  |  |  |  |  |  |  |  |  |  | Outdoor | **0.74[0.66-0.84]** | **<0.001** |  |
|  | Microscopy | Cleaner | *Ref.* |  |  | Charcoal | *Ref.* |  |  | Indoor | *Ref.* |  |  |
|  |  | Biomass | **4.72[3.67-6.06]** | **<0.001** | 23519 | Wood | **1.77[1.57-2.00]** | **<0.001** | 35898 | In a separate building | **0.66[0.59-0.73]** | **<0.001** | 18209 |
|  |  |  |  |  |  |  |  |  |  | Outdoor | **0.84[0.74-0.95]** | **0.005** |  |
| Wood only | RDT |  |  |  |  |  |  |  |  | Indoor | *Ref.* |  |  |
|  |  |  |  |  |  |  |  |  |  | In a separate building | **0.66[0.59-0.74]** | **<0.001** | 19406 |
|  |  |  |  |  |  |  |  |  |  | Outdoor | **0.77[0.68-0.87]** | **<0.001** |  |
|  | Microscopy |  |  |  |  |  |  |  |  | Indoor | *Ref.* |  |  |
|  |  |  |  |  |  |  |  |  |  | In a separate building | **0.63[0.56-0.72]** | **0.001** | 17244 |
|  |  |  |  |  |  |  |  |  |  | Outdoor | **0.86[0.75-0.99]** | **0.03** |  |
| **Exploratory analysis** | | | | | | | | | | | | | |
| Controlling for household mosquito spraying† | RDT | Cleaner | *Ref.* |  |  | Charcoal | *Ref.* |  |  | Indoor | *Ref.* |  |  |
|  |  | Biomass | **4.70[3.59-6.15]** | **<0.001** | 26778 | Wood | **3.88[3.32-4.53]** | **<0.001** | 36898 | In a separate building | **0.67[0.58-0.77]** | **<0.001** | 9951 |
|  |  |  |  |  |  |  |  |  |  | Outdoor | **0.73[0.63-0.85]** | **<0.001** |  |
|  | Microscopy | Cleaner | *Ref.* |  |  | Charcoal | *Ref.* |  |  | Indoor | *Ref.* |  |  |
|  |  | Biomass | **4.80[3.58-6.45]** | **<0.001** | 18102 | Wood | **2.20[1.91-2.53]** | **<0.001** | 27115 | In a separate building | **0.59[0.51-0.69]** | **<0.001** | 9676 |
|  |  |  |  |  |  |  |  |  |  | Outdoor | 1.00[0.87-1.15] | 0.99 |  |
| Abbreviation: AOR = Adjusted Odds Ratio, 95% CI = 95% confidence interval, N= Number of observations, RDT = Rapid diagnostic test. Ref = Reference group. Results in bold are statistically significant.  *Controlled for: Child’s age, child’s gender, birth order, Child slept under slept under mosquito net last night, modified wealth index, number of household members, place of residence, malarial endemicity, dwelling construction, season and cluster altitude.  † Burkina Faso 2017-18, Cameron 2018, DRC 2013-14, Malawi 2017, Mali 2018, Nigeria 2018, Tanzania 2017 and Togo 2017 were excluded due to the household mosquito spraying variable being incomplete, high level of missing or low cell counts. | | | | | | | | | | | | | |
